# Pediatric Genitourinary Oncology

**DOI:** 10.3389/fped.2013.00048

**Published:** 2013-12-16

**Authors:** Francisco Tibor Dénes, Ricardo Jordão Duarte, Lílian Maria Cristófani, Roberto Iglesias Lopes

**Affiliations:** ^1^Uropediatric Unit, Division of Urology, Hospital das Clínicas, University of São Paulo, São Paulo, Brazil; ^2^Pediatric Onco-Hematology Unit, Department of Pediatrics, Hospital das Clínicas, University of São Paulo, São Paulo, Brazil

**Keywords:** pediatric tumor, kidney neoplasms, rhabdomyosarcoma, testicular neoplasms, adrenal tumors

## Abstract

Tumors of the kidney, bladder, prostate, testis, and adrenal represent a large part of the adult urologic practice, but are relatively infrequent in children. The natural history and management of these tumors in the pediatric age is different from that of the adults. As result of the successful work of several clinical trial groups in recent decades, there has been a significant improvement in their cure rates. The aim of this article is to review their most significant clinical aspects, as well as to present an update in their management.

## Kidney Tumors

Among the primary pediatric kidney tumors, the most frequent is the Wilms tumor (WT), followed by the mesoblastic nephroma. The incidence of the different tumors is shown in Table 1 (1, 2).

**Table 1:** **Pediatric kidney tumors: classification and frequency (1)**.

Histological type	Frequency (%)
Wilms’ tumor	85
Mesoblastic nephroma	5
Clear cell sarcoma	4
Rhabdoid tumor	2
Miscellaneous	4

### Wilms tumor

Wilms tumor or nephroblastoma is the most common genitourinary malignant tumor of children. The incidence in the United States is seven per million children below 15 years of age, while in different regions of Brazil it varies from 4 to 15 cases per million per year. The peak incidence occurs between 2 and 3 years of age (3, 4).

Mutations or deletions of the *WT1* gene of the 11p13 locus are present in 15–20% of sporadic tumors, while the *WTX* gene shows mutations in 29% of them. Loss of heterozygosity (LOH) of the 1p and/or 16q chromosomes is a factor of adverse prognosis (5). Loss of imprinting (LOI) or LOH at 11p15 leads to *IGF2* overexpression and is present in 70% of WT patients. Some syndromes and conditions are associated to *WT* gene mutations and to the presence of intralobar and perilobar nephrogenic rests, leading to an increased risk of developing WT, such as Beckwith–Wiedemann, Denys–Drash, WAGR, and corporal hemihypertrophy (6, 7).

The majority of WT is characterized by a triphasic histology, that includes blastematous, epithelial, and stromal components. The most important histological factor in prognosis is the occurrence of anaplasia, which is observed in 5% of the cases and is characterized by the presence of multiple mitoses, as well as increase in size and hyperchromasia of the cell nucleus. Its presence, either focal or diffuse, indicates an increased aggressiveness of the tumor, even if it is still localized, as in stages I and II (8).

In general, WT manifests itself as an asymptomatic abdominal mass, which is palpated by the parents or caretakers in 90% of the cases. Macroscopic hematuria occurs in 25% of cases. Abdominal pain is referred in 30%, while high blood pressure is observed in about 25% of the patients. Up to 12% of patients with WT have associated congenital abnormalities, such as cryptorchidism, hypospadias, hemihypertrophy, and aniridia (9). Between 5 and 15% of patients have bilateral or multicentric tumors. The average age at presentation is 42–47 months for patients with unilateral tumors and 30–33 months for those with bilateral tumors (7).

Usually, the abdominal ultrasound (US) examination confirms the presence of a renal mass, but computer tomography (CT) is used to provide all information required for adequate diagnosis and staging of this tumor, including infiltration of adjacent structures, extension to the inferior vena cava or abdominal or thoracic metastases (7). In some cases, Doppler US or magnetic resonance imaging (MRI) may be necessary to confirm involvement of the renal veins and cava. Differential diagnosis includes neuroblastoma, which often occurs in children of the same age. The staging system for WT is presented in Table 2 (10).

**Table 2:** **Wilms tumor staging system (10)**.

I – tumor restricted to the kidney, completely excised, no penetration of the capsule or involvement of renal sinus vessels
II – tumor beyond the kidney, but without residual beyond the margins of excision. Eventual tumoral thrombus outside the kidney, but completely removed en-bloc with the tumor
III – gross or microscopic residual local tumor postoperatively, including positive margins, inoperable tumor, intra-abdominal lymph node metastasis, peritoneal implants, tumoral rupture, or transected tumoral thrombus
IV – hematogenic metastases (liver, lung, bone, brain) or extra-abdominal lymph node metastasis
V – bilateral tumor

Treatment of WT includes surgery, chemotherapy, and in some patients radiotherapy. There are two treatment protocols for the initial management of this tumor. One, recommended by the Children’s Oncology Group (COG) advocates up-front surgical removal without interference of neoadjuvant antineoplastic drugs, thus providing a detailed histological staging and accurate cytogenetic studies, necessary for planning post-operative treatment. The other protocol is proposed by the International Society for Pediatric Oncology (SIOP), which advocates 4–6 weeks of chemotherapy before nephrectomy, with or without concomitant metastases. Despite the potential adverse effect of neoadjuvant chemotherapy in staging and histologic evaluation, its beneficial effects are the reduction of the tumoral size and the formation of a fibrous pseudocapsule that facilitate the surgical removal and decrease the risks of rupture and spillage during surgery.

In both protocols, chemotherapy is based on vincristine and dactinomycin for stage I and II with favorable histology and vincristine, dactinomycin, and doxorubicin for stage III and IV with favorable histology. Treatment of tumors with anaplastic histology, advanced stages (III and IV), or greater-risk tumors also includes cyclophosphamide or ifosfamide, carboplatin, and etoposide, eventually associated to radiation therapy (7). Stage V disease (bilateral tumors) requires preoperative chemotherapy with vincristine, dactinomycin, and eventually doxorubicin for 8 weeks, followed by nephron sparing surgery. In the long-term follow-up, the survival rates of both strategies, based in the surgical removal, pre and/or post-operative chemotherapy and radiotherapy, are equivalent (10, 11). The current trials of both protocols are trying to minimize the late effects of treatment without compromising the excellent overall survival (12).

The classical surgical procedure for the treatment of WT is open radical nephrectomy through a transperitoneal access (10, 13). The adrenal glands may be left “*in situ*,” if they are unchanged and can be easily separated from the kidney; otherwise, they are removed. Although extensive lymphadenectomy is not required, perihilar and interaortocaval lymphnode samples must be obtained in all cases, as they are necessary to adequate staging and planning of post-operatory management. Failure to remove and evaluate lymphnodes increases the risk of local recurrence and significantly decreases the 5-year survival rate (9, 14).

According to the consolidated experience in the treatment of adult renal tumors, the well-known benefits of laparoscopic radical nephrectomy, namely reduced hospitalization and analgesic requirement, as well as the better cosmetic result, were extended to selected patients with WT. Although still considered experimental, this technique has been employed with good results in several centers in the treatment of unilateral tumors, with or without neoadjuvant chemotherapy, without impairing the oncological results (15–17).

Recent consideration has been given to nephron sparing surgery in selected patients with unilateral WT. While long-term, prospective data is not yet available, case series demonstrate preservation of renal function and comparable oncologic outcomes after partial vs. radical nephrectomy (18, 19).

In 5–7% of the cases, WT involves both kidneys. In this scenario, besides complete removal of the tumor masses, treatment also aims preservation of as much healthy renal tissue as possible, by means of nephron sparing surgery (20). Preoperative tumor biopsy is not always required. Neoadjuvant chemotherapy is recommended for 6–12 weeks, after which the patient is re-evaluated by CT in order to plan the best surgical approach. Possible alternatives are bilateral partial nephrectomy or unilateral radical nephrectomy associated to contralateral partial nephrectomy. The risk of end-stage renal disease in bilateral WT is 5.4–12%, compared to 0.2–0.6% in unilateral tumors (12). Bilateral radical nephrectomy is very rarely employed, as it implies immediate dialysis and later renal transplantation.

In 4–10% of cases, WT may present with tumoral thrombus in the renal vein, vena cava, or atrium, diagnosed by Doppler US, CT, or MRI (10, 13). If the tumor thrombus is above the hepatic veins, neoadjuvant chemotherapy is required to reduce the size of the tumor and of the thrombus. Those of the inferior vena cava may be extracted together with the tumor by incision of the vessel below the diaphragm. For those that reach above the diaphragm, thoracotomy, and cardiotomy with extracorporeal circulation is necessary.

Post-operative treatment depends on the stage of the disease, as well as the histological evaluation of the specimen and the lymphnodes obtained in surgery. As a rule, patients receive adjuvant chemotherapy in all stages, with the eventual exception of children younger than 2 years of age with stage I disease and tumors with favorable histology weighing <550 g. All stage III patients receive local irradiation, while in case of major tumoral rupture a whole abdominal radiotherapy is necessary (11). In the COG protocol, all patients with anaplastic histology also require radiotherapy (10, 11).

The 5-year event-free survival is estimated in 87% for stage I and 85–74% for stage II, 82% for stage III, and for stage IV it decreases to 60–70% (21). Recurrence in patients with stage I or II is associated to significant mortality even in favorable subtypes, with overall survival dropping to 50% despite the use of more intense chemotherapy in these relapsed cases (12).

Although significant success has been achieved in increasing the overall 5-year survival rates to more than 90%, multimodal therapy is associated to late adverse effects, which require long-term monitoring of these patients (12). Around 0.7% of patients with unilateral WT will develop renal failure, an incidence eightfold higher than that expected in the general population (22). Fifteen years after diagnosis, the cumulative incidence of a second malignancy is 1.6%, with risk factors including abdominal radiotherapy as part of the initial or adjuvant treatment, with or without chemotherapy for relapse (23). In the NWTSG Late Effects Study, the cumulative incidence of congestive heart failure (CHF) 20 years after diagnosis of WT was 4% in patients whose treatment plan included doxorubicin, with a direct dose–response relationship (each 100 mg/m^2^ of doxorubicin exposure increased the relative risk of CHF by 3.3). Furthermore, patients treated for relapsed WT had a higher cumulative frequency of CHF compared with patients without relapse (24).

### Mesoblastic nephroma

Congenital mesoblastic nephroma (CMN) is the most common solid kidney tumor of the newborns, also called renal hamartoma or leiomyomatous hamartoma. Usually, this tumor manifests itself in the first 3 months of life, with a slight predominance in males, as a painless abdominal mass. In some cases, it may be detected in the antenatal US, accompanied by polyhydramnios and hydropsy. It is also associated to early delivery.

At the time of detection, almost all CMNs are predominantly localized in the kidney and perinephric or hilar soft tissue. The imaging studies show a large, solid intrarenal mass that involves the sinus, generally presenting cystic, hemorrhagic, and necrotic areas (2). Histopathologically, they are predominantly monomorphic neoplasms composed of spindled mesenchymal cells of fibroblastic or myofibroblastic lineage. These are divided into two major types: classic and cellular. Cytogenetic evaluation of this tumor has shown that the cellular type is almost invariably associated with abnormalities of the *t*(12;15) and 11+, whereas the classical type rarely is. These abnormalities are also observed in congenital fibrosarcoma (25).

Treatment is based on radical nephrectomy, and free margins are necessary due to the infiltrative tendency of this tumor. Prognosis is usually good, particularly in cases treated before the sixth month of life Recurrences and metastasis occur in approximately 5–10% of all tumors (26).

### Rhabdoid tumor

This is a rare tumor, with 80% of cases occurring in children before 2 years of age, mainly in boys, with a proportion to girls of 1.5:1 (2, 10). Some patients present synchronous brain lesions or atypical teratoid/rhabdoid tumor (AT/RT) that resemble primitive neuroectodermal tumors, and have the same genetic mutation (*SMARCB1*) found in the renal lesion (26). Approximately one-third of newly diagnosed patients with RT have an underlying genetic predisposition to tumors due to a germline SMARCB1 alteration. Family members may demonstrate incomplete penetrance and gonadal mosaicism, which must be considered when counseling parents of patients with RT (27).

The common symptom is hematuria, but often there are signs and symptoms related to metastatic spread to the lung, liver and brain, that are present at the time of diagnosis in up to 80% of the cases (2, 26). Preoperative biopsy is eventually required for pathological diagnosis, but early radical nephrectomy is necessary for cure. It is a very aggressive tumor, usually resistant to chemotherapy and radiotherapy. The 4-year survival rate varies from 20 to 36% (10, 28).

### Clear cell sarcoma

This tumor has a peak incidence between 1 and 4 years of age, with a predominance of boys over girls of 2:1. There are no known familiar or syndromic associations, nor bilateral cases (2, 16). The main sign is a palpable abdominal mass, and 15–60% of the cases present skeletal metastases at the time of diagnosis, with pain as the main symptom. The imaging studies do not allow differentiation from WT. This tumor is sensitive to chemotherapy, therefore it should be associated to radical nephrectomy as the treatment of choice. The 5-year relapse-free survival and overall survival rates reach as high as 79 and 86% respectively, for patients receiving 28–34 weeks of chemotherapy (28, 29).

### Renal cell carcinoma

Renal cell carcinoma (RCC) is the diagnosis in 2–5% of the pediatric renal tumors. Between 0.5 and 2% of all RCC occur in patients younger than 21 years of age, mainly between 9 and 15 years (2, 10, 26). It may be associated to the von Hippel–Lindau syndrome, in which the tumors are multiple and have an earlier occurrence (2). One population based study showed about 30% of cases associated to underlying disorders, like tuberous sclerosis, neuroblastoma, teratoma, Saethre–Chotzen syndrome, chronic renal failure, or related diseases in their family (30).

Clinical presentation is similar to that of adults, with painless macroscopic hematuria, flank pain, and palpable mass being the most frequent symptoms. In general, RCC is smaller than WT at the time of diagnosis. CT study shows a solid non-enhanced intrarenal lesion, with areas of hemorrhage, necrosis, and calcification, but clear differentiation from WT is not possible (2). At presentation, more than two thirds of the patients have localized disease. In metastatic disease, two thirds are in the regional lymph nodes and one-third in distant organs, mainly lung, liver, and brain (2, 30).

Childhood RCC differs histologically and biologically from those of adults. The papillary form occurs in 20–50% of the cases, while the rest is represented by the classical clear cell carcinoma (10, 26). Genetic translocations are found in 30% of the RCC in children, most of them involving the *TFE3* gene on chromosome X p 11.2. Other biological targets of therapeutic interest may be present and include *c-Met, mTOR*, and *VEGRF*. Morphologically, these carcinomas resemble the conventional clear cell tumor, but present areas of papillary architecture. They represent a distinct class of renal carcinoma in the WHO classification (2, 10). Treatment consists in the complete removal of the tumor by radical nephrectomy, either by open or laparoscopic access. Lymphnode sampling is recommended for adequate staging. The adrenal gland may be preserved in cases of lower pole tumor. Initial experience suggests that equivalent cure rates can be expected from a nephron sparing approach in appropriately selected cases (31). This tumor is resistant to chemotherapy or radiotherapy, and the treatment of metastases is still a challenge. Angiogenesis-inhibiting or immune-stimulating agents, like vaccines, interferon, sorafenib, sunitinib, and sirolimus, are possible therapeutic alternatives, with limited temporary results. Event-free survival and overall survival rates at 5 years reach 96% for localized RCC, 69 and 75% for regional lymph node-positive patients, 25 and 33% for patients with distant metastases, respectively (30). Recent studies show that survival in children with lymphatic spread only is better than in adults (10, 28).

### Medullary carcinoma

Described recently in 1995, it affects carriers of sickle-cell disease trait. It presents a very aggressive behavior, with frequent metastases and a mortality rate close to 100%. Treatment is based on radical nephrectomy, as it is unresponsive to chemo or radiotherapy (28).

Other less common renal malignancies of childhood include the anaplastic sarcoma, cystic nephroma, primitive neuroectodermal tumor, desmoplastic small round cell tumor, as well as the intrarenal neuroblastoma. Adenomas, lipomas, lymphomas, and angiomyolipomas are also described in children. In these, as in the previously described tumors the preoperative imaging studies are mostly unable to establish the preoperative diagnosis. Their treatment is mostly based on radical nephrectomy (12).

## Rhabdomyosarcoma

Rhabdomyosarcoma (RMS) is a malignant tumor that originates from the embryonic mesenchymal cells that give origin to the striated musculature. It may surge in various sites in the body, even where the striated muscle is not usually present. They represent 4–8% of the malignant tumors in patients younger than 15 years of age, and around 21% of them rise in the genitourinary tract, mainly in the bladder, prostate, vagina, and the paratesticular region (32).

The annual incidence in the United States is 4.5 cases per million children younger than 15 years of age. There is a bimodal age distribution with a peak incidence in the first 2 years of life and another in adolescence. Etiology is unknown, but a possible explanation is the regulatory disruption of striated muscle progenitor cell growth and differentiation (33). Some environmental factors are associated to an increased risk for developing the disease, like paternal smoking habit, advanced maternal age, intrauterine exposition to radiation, and use of illicit drugs by the mother. A genetic predisposition is found in the Li–Fraumeni syndrome and neurofibromatosis. Compared to the general population, patients with RMS have an increased incidence of associated congenital malformations (32 vs. 3%) (34, 35).

Rhabdomyosarcoma includes a heterogeneous group of tumors, that present two main histologic variants: the embryonal, which is predominant, and the alveolar. The embryonal RMS is subdivided into spindle cell and the botryoid sarcoma subtypes. Spindle cell histology is common in paratesticular lesions, whereas botryoid lesions are polypoid masses that fill the lumen of the bladder and vagina (33, 35). Embryonic RMS generally presents a favorable prognosis. Alveolar histology, which is characterized by two genetic reciprocal translocations *PAX3-FOXO1 and PAX7-FOXO1*, is more prevalent in adolescents and confers a lesser chance of cure due to the high rate of local recurrence (32, 34, 36).

The clinical symptoms of genitourinary RMS depend on the site of the lesion. Paratesticular RMS presents as unilateral painless scrotal mass distinct from the testis, which is fortuitously palpated. At diagnosis, retroperitoneal lymph node extension is detected in 20–40% of these patients. Bladder or prostatic tumors generally cause voiding symptoms like frequency, dysuria and hematuria, but urinary retention is the most frequent. On physical examination, a pelvic mass can be palpated. Girls with primary tumor in the vagina or uterine cervix may present in the first years of life with vaginal bleeding or discharge and occasional exteriorization of the polypoid mass through the vulva (34, 35).

Patients with suspected bladder, prostate, and vaginal tumors require complete laboratorial and imaging studies. MRI provides excellent definition of the primary tumor and surrounding tissues, while CT is useful for evaluation of bone erosion and abdominal lymphadenopathy. Bone scan is necessary for the search of skeletal metastases, while the PET-scan is useful in the evaluation of the primary lesion as well as regional and distant metastases (33, 37). Diagnosis is confirmed by biopsy or resection of the tumor.

Paratesticular tumors are best managed initially by radical orchiectomy through the inguinal approach.

According to the recommendations of the Intergroup RMS Study Group, most boys older than10 years of age should then undergo staging ipsilateral retroperitoneal template lymph node dissection followed by multiagent chemotherapy and radiotherapy. In children aged <10 years, however, PET-CT may be sensitive for the identification of metastatic retroperitoneal lymphatic metastases, thus reducing the need of lymphadenectomy (38). Radiotherapy is beneficial in patients with lymph node-positive disease (39).

Bladder or prostatic lesions require cystoscopic evaluation and biopsy, but when these are unsuccessful, needle biopsy or open surgery may be necessary. Protruding vaginal masses are easily biopsied. All samples must be submitted to histopathological, immunohistochemical, cytogenetic, and molecular studies. Presently, the main therapeutic objective in vesico-prostatic or vaginal-uterine RMS is to ensure cure with maximal functional and anatomical preservation of the involved organs, avoiding mutilating procedures that are associated to increased morbidity. After imaging and pathological studies, neoadjuvant chemotherapy is initiated. With size reduction of the tumor, definitive but conservative surgical resection is recommended. The role of surgery is decisive in the success of the treatment, since local control of the disease correlates with prognosis. Complete resection of the primary tumor is ideal. Microscopic evaluation of the surgical margins, as well as lymphnode sampling for staging, is highly recommended. Adjuvant chemo and radiotherapy are then initiated according to the stage of the disease and the success of surgery (40).

Rhabdomyosarcoma is staged by the Soft Tissue Sarcoma Committee with a disease-specific TNM staging system (Tables 3 and 4) (41). Considering the stage, group and histology, patients are assigned to low, intermediate, or high-risk groups. Those with paratesticular and vaginal RMS with embryonic histology and complete resection, even with microscopic residual, are considered low-risk patients. Intermediate-risk patients include bladder and prostatic RMS, as well as those with gross residual disease. High-risk patients are those with metastatic disease (40–42).

**Table 3:** **Preoperative staging of rhabdomyosarcoma (41)**.

Stage	Organ	Tumor (T)	Size	Lymphnode (N)	Metastases (M)
I	Paratesticular, vaginal uterine	T1 or T2	a, b	N0, N1, Nx	M0
II	Bladder, prostate	T1 or T2	a	N0, Nx	M0
III	Bladder, prostate	T1 or T2	a	N1	M0
	b	N0, N1, Nx	
IV	All	T1 or T2	a, b	N0 or N1	M1

*T1, tumor confined to site of origin (a, diameter <5 cm; b, diameter >5 cm)*.

*T2, local infiltration, extension, or adherence (a, diameter <5 cm; b, diameter >5 cm)*.

*N0, negative regional lymph nodes; N1, positive regional lymph nodes*.

*Nx, lymph nodes status unknown; M0, no distant metastases; M1, positive distant metastases*.

**Table 4:** **Post-operative classification of rhabdomyosarcoma (41)**.

Group 1	Localized disease, completely removed, without microscopic residual
A	Confined to the site of origin, but completely removed
B	Infiltration beyond site of origin, but completely removed
Group 2	Total macroscopic resection
A	Macroscopic resection with evidence of microscopic residual lesion
B	Regional disease with lymph node extension, completely resected without microscopic residual lesion
C	Local microscopic residual lesion and/or residual lymph node extension
Group 3	Incomplete resection or biopsy, with residual mass
Group 4	Distant metastases

Patients with positive lymph nodes, also when detected by PET-CT, must be submitted to more intensive chemotherapy and eventual radiotherapy. This multimodal approach ensures survival rates of 90% (35, 43). In tumors that involve the bladder and prostate, around 70% are originated in the bladder. In most of these cases, neoadjuvant chemotherapy and radiation allow a bladder sparing surgery. It is possible to achieve disease-free survival with preservation of vesical function by partial cystectomy in 50–60% of cases, particularly in those with tumors arising in the bladder dome. Larger tumors, as well as those originating from the bladder base or prostate and also those resistant to neoadjuvant chemotherapy eventually require extensive resection with ureteral reimplantation, cystectomy, or cystoprostatectomy, with need of urinary diversion (44). In general, tumors that originate from the vagina respond well to chemotherapy, rarely requiring procedures like partial vaginectomy or vaginectomy with hysterectomy. Late complications like vaginal stenosis, ureteral obstruction, intestinal stricture or fistula, and ovarian failure are often observed after multimodal therapy (42).

Chemotherapy is based in the use of vincristine, actinomycin D, and cyclophosphamide for low-risk patients. The group of intermediate-risk may receive the same drugs associated to ifosfamide or etoposide. High-risk patients must be treated with a combination of ifosfamide, etoposide, and doxorubicin. The combination of vincristine and irinotecan is useful in metastatic disease. The aim-therapy for *mTOR* receptors, employing rapamycin, temsirolimus, and everolimus, as well as IGF-1R, is still under investigation. Children with low-risk tumors have 5-year survival rates of 90%, while those with intermediate-risk reach 55–70% and those with high-risk <50% (41–43, 45).

## Transitional Cell Carcinoma

Transitional cell carcinoma of the bladder in children is very rare, with about 150 patients younger than 20 years presented in the literature, only 20% of them in the first 10 years of age. It predominates in white children with a male-to-female ratio of 3:1 (46–48).

Asymptomatic macroscopic hematuria is the most common onset symptom. Imaging studies with US followed by cystoscopy are the ideal diagnostic tools. There are few isolated reports of upper urinary tract involvement, therefore the kidneys must always be evaluated by ultrasound. Most lesions are located in the trigone, and the predominant histological pattern is papillary and non-invasive. Endoscopic resection is the standard treatment, being effective in all cases of localized disease. Follow-up includes regular ultrasound evaluation and urinary cytology every 6 months in the first 2 years and cystoscopy once a year. Recurrence is described in 13% of the patients, and progression to invasive disease with muscular involvement occurs in 10–15% of cases (48, 49).

## Testicular Tumors

Testicular tumors are less frequent in childhood than in post-pubertal age. The annual incidence is 5.9/100,000 boys younger than 15 years of age, with the peak incidence in the first 3 years of life. They represent 2% of the pediatric tumors, and two thirds of them are of benign nature (Table 5) (50, 51).

**Table 5:** **Relative frequency of the different tumor types registered in the “prepuberal testis tumor registry” (52)**.

Type of tumor	*N*	%	Behavior
Yolk sack tumor	244	62	Malignant
Teratoma	92	23	Benign
Indifferentiated stromal	16	4	Occasionally malignant
Epidermoid cyst	13	3	Benign
Juvenile cell of granulosa	11	3	Benign
Sertoli cell	10	3	Malignant in older children
Leydig cell	5	1	Benign
Gonadoblastoma	4	1	Usually benign

Increase in the scrotal volume and palpation of a hardened painless testicular mass are the initial findings in 88% of the patients. In some cases, a hydrocele or hernia may lead to the diagnosis. Inflammatory processes, as well as cysts and particularly testicular torsion must be excluded (53). Physical examination is important, in order to evaluate signs of virilization. The preferred imaging modality for these tumors is doppler US, that identifies the presence, size, and exact location of the mass, but does not differentiate between benign and malignant lesions. Nevertheless, some tumors have specific US characteristics, like the epidermoid cysts and the teratoma. Alpha-feto-protein (AFP) is an important tumor marker, and has increased blood levels in most cases of malignant prepuberal testicular tumors. Human chorionic gonadotropin (β-HCG) blood levels are rarely increased in these tumors. In confirmed malignant tumors, it is necessary to search for retroperitoneal and pulmonary metastases with CT imaging. Staging is based in image studies and tumoral markers, as well as the pathological findings (Table 6).

**Table 6:** **Children’s Oncology Group (COG) staging system for malignant testicular tumors of childhood (53)**.

Stage	Extent of disease
I	Limited to testis, completely resected by high inguinal orchiectomy. Absence of clinical, radiographic, or histologic evidence of residual disease
II	Transcrotal biopsy, microscopic disease in scrotum or in spermatic cord (<5 cm from proximal end). Tumor markers fail to normalize or decrease with appropriate half-life
III	Retroperitoneal lymph node involvement, but no visceral or extra-abdominal involvement. Lymph nodes >4 cm by CT, or >2 cm and <4 cm with biopsy proof
IV	Distant metastases, including liver

Surgical treatment usually begins with radical orchiectomy, which is recommended whenever the AFP is elevated. It is performed through an inguinal incision with control of the vessels at the internal inguinal ring and removal of the testis without violating the vaginal tunic. If the AFP is normal, the tumor is probably benign, as in cases of teratoma and epidermoid cyst. A partial orchiectomy may be then considered, requiring protection of the operative field, clamping of the pedicle, incision of the vaginal tunic and albuginea, and careful enucleation of the mass. The tunics are then closed, and the testis reinserted in the scrotum after clamp removal (52, 54).

In the post-operative period, the patient is observed in case of benign tumors. In case of malignancy with stage I testes tumors, surgery alone presents excellent survival rates, with recurrence in 15.5% of the patients. Stage II disease is associated with 75% recurrence rate. In both cases, recurrence is successfully treated with chemotherapy based on cisplatin, etoposide, and bleomycin. Higher stages of the disease have also excellent prognosis, with association of surgery and chemotherapy (52).

### Yolk sack tumor

It is the most common testis tumor in children younger than 2 years of age, presenting as a solid testicular mass associated to increased levels of AFP (51). Most cases present with localized disease, and only 4–6% have retroperitoneal or pulmonary metastases, which are suspected by persistently increased levels of AFP after orchiectomy and evidenced by imaging studies.

Radical orchiectomy is generally curative in localized disease, therefore if the post-operative blood levels of AFP, whose half-life 5–7 days, are normal and the imaging studies are normal, there is no need of adjuvant therapy (55). Cases with persistently elevated APF or confirmed metastases must be treated with chemotherapy, presenting 6-year survival rates near 100% (55–57).

### Teratoma

It is the second most frequent prepuberal testicular tumor, with a peak incidence at 13 months of age (55). Contrary to those that occur in the adult age, prepuberal teratomas are usually benign (58). Although not specific, the US finding of cysts within the tumor may suggest the diagnosis. As mentioned before, conservative surgical treatment is recommended in most cases, with careful resection of the nodule and preservation of the normal testicular tissue. However, in patients with signs of virilization, radical orchiectomy is indicated (59).

### Epidermoid cyst

These lesions are composed entirely of keratin producing epithelium, which is easily identified in most cases by US evaluation, due to the peculiar aspect of concentric layers similar to that of an onion (60). This tumor probably represents a monodermal teratoma. It is usually benign and is best treated with conservative surgery (50, 51, 54).

### Stromal tumors

These are very rare tumors in this age group. They are represented by the following four lineages (61):

– *Leydig cell tumor*: usually benign, it occurs between 5 and 10 years of age and can manifest with virilization. It may be treated with partial orchiectomy.
– *Sertoli cell tumor*: occurs between 4 months and 10 years of age. Although hormonally inactive in most cases, it can occasionally cause gynecomasty or early puberty. There are no reports of metastases in this age group, but older boys can present disseminated disease. Accordingly, simple orchiectomy may be curative in younger patients, but in older boys imaging studies are necessary to identify metastatic disease.
– *Tumor of the juvenile cell of the granulosa*: it is named by the presence of cells that resemble the granulosa layer of the juvenile ovary. Occurs mainly in the first semester of life and is associated to abnormalities of the Y chromosome. There may be an association with genital ambiguity. The usual treatment is simple orchiectomy.
– *Undifferentiated stromal tumor*: it presents areas of stromal neoplasia associated to undifferentiated areas of fusiform cells with high degree of mitoses. Most cases are benign, but in older boys they may present malignant behavior. Routine treatment is orchiectomy, but adjuvant chemotherapy may be necessary in cases of confirmed malignancy.

### Gonadoblastoma

Patients with disorders of sexual differentiation present an increased incidence of gonadal tumors in the presence of the Y chromosome, particularly when associated to intra-abdominal cryptorchism or disgenetic gonads. Although more frequent in the post-puberal age, they may occur in childhood. They are usually benign and asymptomatic, but may be associated to virilization. If not removed, they may progress to disgerminomas, that present malignant behavior. Prophylactic laparoscopic gonadectomy is recommended in these cases (61, 62).

## Adrenal Tumors

The adrenal gland may be site of several types of tumors, either benign or malignant, primary, or metastatic. Primary adrenal tumors originate from the medulla or cortex, and can be hyper or non-functioning. The group of primary medullary neoplasms include the neuroblastoma, ganglioneuroblastoma, and ganglioneuroma, which are collectively referred as neuroblastic tumors, as well as the pheochromocytoma (63). Tumors originating from the cortex include the adrenocortical adenoma and carcinoma. In children, the most common lesions are neuroblastomas and cortical adenomas, with pheochromocytomas and cortical carcinomas occurring more rarely (64).

Diagnosis of adrenal tumors is suspected in children with hormonal abnormalities. Routine US imaging may disclose non-functioning tumors in the minority of patients. Clinical evaluation must include imaging and hormonal studies. Abdominal CT or MRI confirm the side, size, and extension of the tumor, as well as the eventual presence of vascular thrombus, infiltration of adjacent structures and distant metastases.

### Neuroblastic tumors

The adrenal is the most common site of tumors originated from the ganglionar cells. These tumors include the neuroblastoma, which is the most malignant, due to the presence of undifferentiated small round cells, the ganglioneuroma, that is a more mature neoplasm, with a benign behavior, and the ganglioneuroblastoma, which presents mixed histology and has an intermediate behavior. Both neuroblastoma and ganglioneuroblastoma occur more frequently in children, while the ganglioneuroma presents more often in adolescents and young adults (63).

Neuroblastoma is the most common extracranial solid neoplasm in children, arising in the adrenal or in any segment of the sympathetic plexus. In 75% of the cases it is found in the abdomen, being one-third in the medulla of the adrenal gland. It is the most frequent malignant adrenal tumor in childhood, occurring more often during the first 10 years of life, 80% of them in children younger than 5 years of age. It may be diagnosed antenatally or in the first months of life on routine US examinations, but is most often detected as a palpable abdominal mass. Symptoms are related to the mass effect, with pain and abdominal distension, invasion of adjacent organs, metastatic disease or abnormal hormonal production of catecholamines, or vasoactive intestinal polypeptides. Opsomyoclonus or cerebellar ataxia has been observed in up to 4% of patients (63). The tumor consists mainly of primitive neuroblasts, and has a tendency to infiltrate and sometimes invade the adjacent organs and vessels, as well as to present hemorrhage and necrosis. Imaging studies with CT or MRI are necessary to stage the local extension of the lesion, while ^123^I-meta-iodobenzylguanidine scintigraphy is useful to identify distant metastases, mainly to the lymph nodes, bone marrow, liver, and skin, which are present in more than half of the patients at the time of diagnosis (63). Bone metastases are frequently detected in skull, vertebrae, and long bones (63). The prognosis for adrenal neuroblastoma depends mainly on the age of the patient and stage of the disease. In very small children and infants these tumors may spontaneously regress and even disappear (65). Except in this very early age group, in which conservative management may be employed, adrenalectomy is the procedure of choice, either by open or laparoscopic approach. The prognosis is dismal for children older than 18 months of age, with metastatic disease. Tumors presenting amplification of the MYCN oncogene have a bad prognosis and are considered as high-risk group (65). For these patients surgery with chemoradiotherapy, stem cell transplantation, as well as anti-disialoganglioside (GD2) immunotherapy plus cytokines may improve survival (66).

Ganglioneuroblastomas and ganglioneuromas occur more rarely, the former with similar symptoms as the neuroblastoma, the later more often asymptomatically. While differential diagnosis is difficult by imaging studies, urinary catecolamine levels are usually not increased in these tumors (63). Surgical removal is the standard treatment.

### Pheochromocytoma

This neoplasm occurs very rarely in children and represents <1% of all tumors in this age group. It usually occurs in older children, at a mean age of 11 years. Compared to adults, there is an increased tendency of bilaterality, rarely being malignant. Most of these lesions occur sporadically, but a familial heritage is possible, particularly in syndromes like Multiple Endocrine Neoplasia (MEN) type-2 and diseases like von Recklinghausen, von Hippel–Lindau, and Sturge–Weber (63).

Pheochromocytoma generally manifests itself by the increase in blood pressure levels, sweating, tachycardia, headaches, visual blurring, flushing, and diarrhea, secondary to excess production of adrenal epinephrine and norepinephrine, as well as vasoactive intestinal polypeptides. Diagnosis is suspected by the increased seric and urinary levels of cathecolamines and metanephrines, and confirmed by the finding of adrenal masses by US, CT, or MRI. ^123^I-meta-iodobenzylguanidine scintigraphy is useful to exclude multifocal disease. In confirmed cases, initial treatment with alpha- and beta-blockers is recommended to control blood pressure and heart rate. Surgical removal of the tumor must be performed under carefully controlled anesthesia with invasive continuous blood pressure and heart rate monitoring, as well as judicious use of nitroprusside and beta-blockers (63). After removal, intensive hydration is necessary to prevent hypotension. Hypertension and other symptoms usually disappear afterward, but control laboratory exams are necessary to identify residual lesions or recurrence.

Although complete removal of the affected adrenal is the standard procedure, partial laparoscopic adrenalectomy, or tumorectomy have gained acceptance for small tumors, particularly in the hereditary syndromes described before. The obvious advantage of partial adrenalectomy is that it may avoid the need of hormonal replacement in cases of bilaterality, although the risk of recurrence in the remnant tissue is also increased (63, 67). The laparoscopic approach enables both total and partial adrenalectomy also in children (68).

### Adrenocortical tumors

Primary adrenocortical tumors (ACT) are rare in children, with a worldwide incidence of 0.3/1 million per year before the age of 15 years. In southern Brazil the prevalence is 15 times higher than in other parts of the world, due to *TP53 R337H* germline mutations in the population of that area (69, 70). These tumors occur more often before 5 years of age, with a female predominance.

Contrary to adults, in children the ACT are generally detected due to the hormonal abnormalities, including virilization in girls, precocious puberty in boys, hypertension, and less often Cushing’s syndrome. Increased serum levels of adrenal hormones raise the suspicion of ACT, which are confirmed by CT or MRI, that provide information regarding size and local extension of the tumor, as well as vascular invasion and distant metastases (63).

The characteristics of pediatric ACT vary considerably. Laboratory findings, clinical and hormonal features, as well as tumor size are not able to distinguish adenomas from carcinomas before surgery (71). Small homogeneous masses are usually benign while larger masses with areas of hemorrhage, necrosis, and calcification more often present malignant behavior (63). Although it is difficult to define what is a large tumor in a child, functioning tumors that present heterogeneity, infiltration of vessels or neighboring organs, and a slow wash-out in the image studies are to be removed more expeditiously due to increased risk of malignancy.

After clinical investigation, surgical treatment with radical resection is the treatment of choice, and the hormonal disturbances disappear soon after the removal of the affected gland (64). Laparoscopic adrenalectomy has been performed in children with benign ACT with excellent results (68).

Histologic differentiation of ACT in children is more difficult to define than in adults (72, 73). The prognosis is good in young patients with completely removed, non-metastatic, and non-infiltrative lesions. Confirmed cases of carcinoma have an increased risk of recurrence and mortality. COG study ARAR0332 aims to clarify how effective surgery alone is for patients with early stages of ACT, as well as to define the advantages of a more radical surgery with extended lymphadenectomy in selected patients. The efficacy of the combination of mitotane with cisplatin, etoposide, and doxorubicin in advanced stages is also being studied (64, 74, 75).

## Conflict of Interest Statement

The authors declare that the research was conducted in the absence of any commercial or financial relationships that could be construed as a potential conflict of interest.
